# The impact of a standardized micronutrient supplementation on PCOS-typical parameters: a randomized controlled trial

**DOI:** 10.1007/s00404-019-05194-w

**Published:** 2019-05-17

**Authors:** Marlene Hager, Kazem Nouri, Martin Imhof, Christian Egarter, Johannes Ott

**Affiliations:** 1 Clinical Division of Gynecologic Endocrinology and Reproductive Medicine, Medical University of Vienna, Spitalgasse 23, 1090 Vienna, Austria; 2 Department of Obstetrics and Gynecology, Landesklinikum Korneuburg, Korneuburg, Lower Austria Austria

**Keywords:** Micronutrients, Polycystic ovary syndrome, Anti-Mullerian hormone, Testosterone

## Abstract

**Purpose:**

To evaluate whether a micronutrient supplementation preparation that includes a high amount of omega-3 unsaturated acids, other anti-oxidants and co-enzyme Q10 would have an impact on specific serum parameters in women with polycystic ovary syndrome (PCOS).

**Methods:**

The study was designed as a monocentral, randomized, controlled, double-blinded trial, from June 2017 to March 2018 (Clinical Trials ID: NCT03306745). Sixty women with PCOS were assigned to either the “multinutrient supplementation group” (one unlabeled soft capsule containing omega-3 fatty acids and one unlabeled tablet containing folic acid, selenium, vitamin E, catechin, glycyrrhizin, and co-enzyme Q10, for 3 months) or the “control group” (two unlabeled soft capsules containing 200 μg folic acid each, for 3 months). The main outcome parameters were anti-Mullerian hormone (AMH), total testosterone, and androstenedione. In addition, the focus was on luteinizing hormone (LH), follicle-stimulating hormone (FSH), the LH:FSH ratio, sexual hormone-binding globulin (SHBG), and estradiol.

**Results:**

In the multinutrient supplementation group, the LH:FSH ratio (2.5 ± 1.1 versus 1.9 ± 0.5, *p* = 0.001), testosterone (0.50 ± 0.19 versus 0.43 ± 0.15, *p* = 0.001), and AMH (8.2 ± 4.2 versus 7.3 ± 3.6, *p* < 0.001) declined significantly, whereas the other parameters, namely estradiol, LH, FSH, androstenedione, and SHBG remained stable.

**Conclusion:**

A micronutrient supplementation that includes omega-3 fatty acids, folic acid, selenium, vitamin E, catechin, glycyrrhizin, and co-enzyme Q10, given for a minimum of 3 months, is beneficial for women with PCOS in terms of PCOS-specific parameters (LH:FSH ratio, serum testosterone and serum AMH).

## Introduction

Polycystic ovary syndrome (PCOS) is a common endocrine disorder that affects about 6–10% of women of reproductive age [1, 2]. In obese patients, lifestyle interventions are generally considered the first-line treatment. Pharmaceutical treatment options include contraceptive pills and hypoglycemic agents [2]. However, contraindications for the oral contraceptive pills are common in this patient population, especially in overweight women, [3] and hypoglycemics often lead to unpleasant side effects [4, 5]. Thus, it is reasonable that women with PCOS were found to seem unsatisfied with pharmaceutical treatment including oral contraceptives and clomiphene citrate and tended to seek out effective alternative management options [6]. This would obviously include nutritional supplements. However, as reviewed recently, there is insufficient high-quality evidence about the effectiveness of nutritional supplements and herbal medicine for PCOS and its related symptoms. Only some low-quality evidence suggests that PCOS women might benefit from inositols and omega-3 fish oil supplements [7].

However, women with PCOS reveal significantly increased levels of oxidative stress [8] and antioxidants have been claimed to exert positive effects on PCOS [9]. Antioxidants that might positively influence the hormonal profile in PCOS include omega-3 fatty acids [7, 9–14], Vitamin E [11, 13, 14], and selenium [15, 16]. Notably, it has been suggested that the anti-inflammatory action of metformin also contributes to the substance’s positive effect in PCOS [17].

Notably, meta-analyses question the effects of an omega-3 fatty acid-only treatment [18, 19]. This raises the question whether single micronutrient supplementations would be of major impact. We, thus, aimed to test the impact of a standardized, multinutrient supplementation on hormonal profile in PCOS women. We chose a micronutrient supplementation preparation that included a high amount of omega-3 unsaturated acids, other anti-oxidants and co-enzyme Q10—the latter had also been shown to improve inflammation and glucose metabolism parameters in previous studies [20, 21]—for our prospective, randomized, controlled study.

## Materials and methods

### Study population and design

In a monocentral, prospective randomized controlled double-blinded trial, 60 women with PCOS were included from June 2017 to March 2018. PCOS was diagnosed according to the revised Rotterdam criteria [22, 23]. Participants had to be 19–35 years of age and had to suffer either from oligomenorrhea, defined as an interval of ≥ 60 days between the last three menstruations, or complete amenorrhoea for at least 90 days. All included women revealed polycystic ovaries on ultrasound. Women who had undergone any kind of PCO-syndrome-related treatment within 3 months before study initiation were excluded. These included metformin, combined oral contraceptives, cortisol therapy, inositol, ovarian drilling, any kind of ovarian stimulation, and in vitro fertilization.

Patients were randomly assigned to one of the following groups: every day, participants received either (i) “PROfertil^®^ female” (Lenus Pharma GesmbH, Seeböckgasse 59, 1160 Vienna), i.e., one unlabeled soft capsule containing omega-3 fatty acids and one unlabeled tablet containing folic acid, selenium, vitamin E, catechin, glycyrrhizin, and co-enzyme Q10 (see Table 1 for details on doses; multinutrient supplementation group), or (ii) two unlabeled soft capsules containing 200 μg folic acid each or (“Folsäure Kapseln” 400 µg^®^, OTC Produktion und Forschung GmbH, Fischergasse 17, 5020 Salzburg; control group).

**Table 1:** PROfertil^®^ female composition overview

Contents	Amount per day
Soft capsule	
Omega-3 fatty acids	500 mg
Pill	
Folic acid	800 µg
Selenium	70 µg
Vitamin E	30 mg
Green tea extract catechin	4 mg
Glycyrrhizin from licorice extract	12 mg
Co-enzyme Q10	30 mg

There were two study-specific consultations: after including the patient (consultation 1: randomization, distribution of drugs and start of study) and after 90–100 days (consultation 2). During consultation 2, unused study blisters were collected and patients were questioned on peculiarities and possible side effects of the micronutrient supplementation used.

On July 15th, 2017, the study had been approved by the Institutional Review Board of the Medical University of Vienna (IRB number: 1232/2016). The study had been registered on ClinicalTrials.gov (Clinical Trials ID: NCT03306745). All patients signed an informed written consent.

### Parameters analyzed

The main outcome parameters were anti-Mullerian hormone (AMH), total testosterone, and androstenedione. In addition, the focus was on luteinizing hormone (LH), follicle-stimulating hormone (FSH), the LH/FSH ratio, sexual hormone-binding globulin (SHBG), and estradiol. All blood samples were obtained within a maximum of 2 weeks before study initiation (baseline visit) and 90–100 days after beginning supplementation with PROfertil^®^ female or folic acid (3-month follow-up). They were obtained during the early follicular phase visit (cycle days 2–5). If necessary, bleeding was induced using 10 mg of oral dydrogesterone twice a day for 10 days. All serum parameters were determined in the central laboratory of the Medical University of Vienna using commercially available assays.

Moreover, women were explicitly asked for any possible side effects during the study period.

The following additional parameters were also collected as general patient information, i.e., age, body mass index and menstruation history; mean antral follicular count; the HOMA index, calculated as insulin (µU/mL) × glucose (mmol/L)/22.5 [24], with an abnormal result defined as value ≥ 2; as well as baseline thyroid-stimulating hormone (TSH) and prolactin levels.

### Sample size calculation

We assumed a reduction of the mean AMH levels by 2 ng/mL with a standard deviation of 3 ng/mL at an alpha value of 0.05 and a power of 0.90. Accordingly, the paired *t* test would require 26 patients per group. A 15% drop-out rate was assumed. This corresponds to four patients per group. Thus, the final sample size was calculated as 30 patients per group, which led to a total study population of 60.

### Statistical analysis

Categorical variables are presented as absolute numbers and percentages, numerical variables as median and interquartile range. All numerical variables were normally distributed. Baseline patient characteristics and hormonal patterns as well as the changes in hormonal parameters between the baseline and the 3-month follow-up were analyzed using paired *t* tests for numerical parameters and the Chi square test or Fisher’s exact test for categorical variables. Differences in hormonal patterns between the baseline and the 3-month follow-up visits were tested using paired *t* tests. *p* values of < 0.05 were considered statistically significant. Statistical analyses were performed using SPSS 24.0 for Windows (SPSS Inc., 1989–2018).

Randomization was performed using nQuery advisorTM Version 7.0.

## Results

Baseline patient and PCOS-specific characteristics of the multinutrient supplementation and the control groups are shown in Table 2. There were no differences between the groups (*p* > 0.05).

**Table 2:** Basic patient and PCOS-specific characteristics of the PROfertil^®^ female and the folic acid only groups

Parameter	Multinutrient supplementation group (*n* = 30)	Control group (*n* = 30)	*p*
Age (years)	27.7 ± 5.7	29.9 ± 4.9	0.116
BMI (kg/m^2^)	26.2 ± 5.6	25.6 ± 5.4	0.672
Abnormal HOMA index	8 (26.7)	9 (30.0)	1.000
Amenorrhea	12 (40.0)	11 (36.7)	1.000
Mean cycle length (days)	66.1 ± 21.5	68.4 ± 18.4	0.653
Mean antral follicular count	19.3 ± 4.0	18.7 ± 1.5	0.722
TSH (μU/mL)	1.5 ± 0.9	1.7 ± 0.8	0.300
Estradiol (pg/mL)	61.6 ± 34.5	59.0 ± 28.1	0.753
Prolactin (ng/mL)	17.0 ± 11.4	13.3 ± 5.3	0.111
LH (mU/mL)	12.9 ± 6.0	11.1 ± 6.4	0.261
FSH (mU/mL)	5.5. ± 1.9	5.8 ± 1.6	0.473
LH:FSH ratio	2.3 ± 1.1	2.0 ± 1.2	0.106
Testosterone (ng/mL)	0.50 ± 0.19	0.43 ± 0.13	0.137
Androstenedione (ng/mL)	3.43 ± 1.57	3.56 ± 1.25	0.140
SHBG (nmol/L)	47.0 ± 20.1	48.9 ± 33.0	0.788

Details on outcome in terms of PCOS-specific hormonal patterns are provided in Table 3. No differences between the baseline and the 3-month follow-up visits were found for the control group. In the multinutrient supplementation group, the LH:FSH ratio (2.5 ± 1.1 versus 1.9 ± 0.5, *p *= 0.001), testosterone (0.50 ± 0.19 versus 0.43 ± 0.15, *p* = 0.001), and AMH (8.2 ± 4.2 versus 7.3 ± 3.6, *p* < 0.001) declined significantly, whereas the other parameters, namely estradiol, LH, FSH, androstenedione, and SHBG remained stable. When comparing the baseline to follow-up changes in hormonal patterns between the multinutrient supplementation and the control groups, significant differences were found for FSH, the LH:FSH ratio, testosterone, and AMH (*p* < 0.05; Table 4). None of the patients reported any side effects.

**Table 3:** Hormonal pattern of PCOS patients in the course of the study

Parameter	Multinutrient supplementation group (*n* = 28)			Folic acid only group (*n* = 28)		
	Baseline visit	3-month follow-up	*p*	Baseline visit	3-month follow-up	*p*
Estradiol (pg/mL)	60.71 ± 39.60	57.18 ± 26.23	0.201	59.61 ± 29.02	57.50 ± 23.07	0.545
LH (mU/mL)	13.2 ± 6.1	10.7 ± 3.6	0.011	11.2 ± 6.5	10.0 ± 5.3	0.172
FSH (mU/mL)	5.5 ± 1.9	5.8 ± 1.8	0.241	5.9 ± 1.6	5.2 ± 1.4	0.038
LH:FSH ratio	2.5 ± 1.1	1.9 ± 0.5	0.001	2.0 ± 1.2	2.0 ± 0.9	0.744
Testosterone (ng/mL)	0.50 ± 0.19	0.43 ± 0.15	0.001	0.43 ± 0.13	0.44 ± 0.12	0.475
Androstenedione (ng/mL)	3.36 ± 1.61	3.17 ± 1.40	0.282	3.51 ± 1.20	3.53 ± 0.98	0.918
SHBG (nmol/L)	46.4 ± 20.2	48.3 ± 19.2	0.252	44.2 ± 27.3	47.1 ± 26.7	0.223
AMH (ng/mL)	8.2 ± 4.2	7.3 ± 3.6	< 0.001	8.0 ± 4.1	8.0 ± 4.0	0.968

**Table 4:** Mean changes of hormonal patterns between baseline and 3-month follow-up in the PROfertil^®^ female and the folic acid-only groups

Parameter	Multinutrient supplementation group (*n* = 28)	Control group (*n* = 28)	*p*
Estradiol (pg/mL)	− 6.36 ± 25.65	− 2.11 ± 18.19	0.477
LH (mU/mL)	− 2.5 ± 4.8	− 1.3 ± 4.7	0.349
FSH (mU/mL)	0.4 ± 1.6	− 0.8 ± 1.9	0.018
LH:FSH ratio	− 0.6 ± 0.9	− 0.1 ± 0.9	0.016
Testosterone (ng/mL)	− 0.06 ± 0.09	0.01 ± 0.07	0.001
Androstenedione (ng/mL)	− 0.12 ± 1.03	0.01 ± 0.73	0.587
SHBG (nmol/L)	1.8 ± 8.3	-2.5 ± 10.6	0.094
AMH (ng/mL)	− 1.0 ± 1.2	0.0 ± 1.1	0.004

## Comment

This prospective, randomized study on PCOS women demonstrated that the use of a multinutrient supplementation containing omega-3 fatty acids, folic acid, selenium, vitamin E, catechin, glycyrrhizin, and co-enzyme Q10 led to a significant reduction in the LH:FSH ratio, testosterone and AMH, when compared to the use of 400 mg folic acid alone. These data support previous reports about beneficial effects of micronutrients, at least of those with an antioxidant activity, on PCOS-specific hormonal profiles [7, 9–16].

It has to be mentioned that previous meta-analyses questioned the effects of a supplementation with omega-3 fatty acids only [18, 19]. It seems obvious that the positive effect which was seen in the present study might be due to the multinutrient approach. However, this approach makes it impossible to attribute the treatment’s positive impact to one of the ingredients. The included ingredients might affect the specific parameters of PCOS via different mechanisms. While omega-3 fatty acids, selenium, vitamin E, and also co-enzyme Q10, known to scavenge free radicals and inhibit lipid and protein oxidation [25], might have acted as antioxidants and thereby likely influenced the proinflammatory PCOS state [9], licorice with its active component glycyrrhizin has been shown to affect androgen metabolism, probably via blocking the activity of 3-β-hydroxysteroid dehydrogenase (3HSD), 17-hydroxysteroid dehydrogenase (17HSD) and 17–20 lyase [26–33] and stimulation of the aromatase activity [31]. Notably, a lowering effect on ovarian androgens has already been reported for licorice [33], even in PCOS women [32]. Last not least, catechin has been reported to decrease testosterone production, at least in male rats. While the authors concluded that the demonstrated decrease in androgens were due to changes in enzymatic activities [34], an antioxidant mechanism cannot be excluded for our study [35].

Thus, one could raise the issue of the used preparation’s composition. PROfertil^®^ female was chosen, since it was a standardized and commercially available multinutrient supplementation. Taken all these considerations together, the fact that the exact impact of each of the ingredients cannot be assessed in detail needs to be considered a study limitation.

Notably, there was one other important difference between the two groups. Since many women with PCOS suffer from infertility, the recommended standard folic acid supplementation of a daily dose of 400 µg [36] was provided to the control group. However, the multinutrient supplementation group received twice the dose. Notably, high-dose folic acid supplementation has been suggested to lead to an improvement in inflammatory factors, biomarkers of oxidative stress [37] as well as metabolic parameters in PCOS women [38]. This might have also contributed to the beneficial effects observed in the multinutrient supplementation group.

Our results must be interpreted with care due to the following additional considerations: although the study seems well randomized (Table 2), patients could have searched for the actual look of the PROfertil^®^ female soft capsules and the pills, for example on the internet. Since the look of the commercially available preparation differs from that of the folic acid capsules, patients could have identified the control medications. This kind of blinding was not according to standards and could have introduced some kind of bias. Moreover, despite the small sample size to achieve, the study period was quite long. Empirically, this was a result of the delay in standardized PCOS treatment for 90–100 days. Accordingly, repeatedly women were not willing to participate. In addition, no data about patients’ dietary habits were collected. Last not least, one could argue that the power was only sufficient to test an AMH difference of 2 ng/mL, whereas a difference of only 1 ng/mL was found in our study, which still reached statistical significance. However, in the sample size calculation, a much higher AMH standard deviation of 3 ng/mL had been expected in contrast to the actual standard deviation of about 1.2 ng/mL, which explains why the lower mean difference in AMH was still found to be significant.

In conclusion, our data suggest that a micronutrient supplementation that includes omega-3 fatty acids, folic acid, selenium, vitamin E, catechin, glycyrrhizin, and co-enzyme Q10, given for a minimum of 3 months, is beneficial for women with PCOS in terms of PCOS-specific parameters, namely the LH:FSH ratio, serum testosterone and serum AMH. Although the clinical relevance of these results can be challenged, the presented date should encourage future studies about the influence of micronutrients on PCOS, hopefully including data about specific symptoms and infertility treatment outcomes as additional outcome parameters.
